# Salt-Tolerant *Synechococcus elongatus* UTEX 2973 Obtained *via* Engineering of Heterologous Synthesis of Compatible Solute Glucosylglycerol

**DOI:** 10.3389/fmicb.2021.650217

**Published:** 2021-05-18

**Authors:** Jinyu Cui, Tao Sun, Lei Chen, Weiwen Zhang

**Affiliations:** ^1^Laboratory of Synthetic Microbiology, School of Chemical Engineering & Technology, Tianjin University, Tianjin, China; ^2^Frontier Science Center for Synthetic Biology and Key Laboratory of Systems Bioengineering, Tianjin University, Tianjin, China; ^3^SynBio Research Platform, Collaborative Innovation Center of Chemical Science and Engineering, Tianjin, China; ^4^Center for Biosafety Research and Strategy, Tianjin University, Tianjin, China

**Keywords:** cyanobacteria, GG, metabolomic analysis, salt tolerance, small RNA regulation

## Abstract

The recently isolated cyanobacterium *Synechococcus elongatus* UTEX 2973 (Syn2973) is characterized by a faster growth rate and greater tolerance to high temperature and high light, making it a good candidate chassis for autotrophic photosynthetic microbial cell factories. However, Syn2973 is sensitive to salt stress, making it urgently important to improve the salt tolerance of Syn2973 for future biotechnological applications. Glucosylglycerol, a compatible solute, plays an important role in resisting salt stress in moderate and marine halotolerant cyanobacteria. In this study, the salt tolerance of Syn2973 was successfully improved by introducing the glucosylglycerol (GG) biosynthetic pathway (OD_750_ improved by 24% at 60 h). In addition, the salt tolerance of Syn2973 was further enhanced by overexpressing the rate-limiting step of glycerol-3-phosphate dehydrogenase and downregulating the gene *rfbA*, which encodes UDP glucose pyrophosphorylase. Taken together, these results indicate that the growth of the end-point strain M-2522-GgpPS-drfbA was improved by 62% compared with the control strain M-pSI-pSII at 60 h under treatment with 0.5 M NaCl. Finally, a comparative metabolomic analysis between strains M-pSI-pSII and M-2522-GgpPS-drfbA was performed to characterize the carbon flux in the engineered M-2522-GgpPS-drfbA strain, and the results showed that more carbon flux was redirected from ADP-GLC to GG synthesis. This study provides important engineering strategies to improve salt tolerance and GG production in Syn2973 in the future.

## Introduction

Cyanobacteria have attracted significant attention recently for the production of green fuels and chemicals due to their ability to absorb sunlight and CO_2_ as sole energy and carbon sources, respectively (Gao et al., 2012, 2016a,b; Luan and Lu, 2018; Matson and Atsumi, 2018). A recently isolated cyanobacterium, *Synechococcus elongatus* UTEX 2973 (hereafter Syn2973), is characterized by a faster growth rate and more tolerance to high temperature and high light (Mueller et al., 2017; Ungerer et al., 2018b). The doubling time of Syn2973 can reach 1.9 h in BG11 medium at 41°C under continuous 500 μmol photons m^–2^ s^–1^ white light with 3% CO_2_ (Yu et al., 2015), which is very similar to that of the heterotrophic yeast *Saccharomyces cerevisiae* (1.67 h) (Herskowitz, 1988). However, as a freshwater cyanobacterium, Syn2973 is very sensitive to salt stress, and its growth was significantly inhibited by high concentrations of salt (Song et al., 2016; Cui et al., 2020b). Meanwhile, large-scale cultivation of cyanobacteria with seawater is necessary for industrial biotechnological applications (Silkina et al., 2019; Cui et al., 2020a). It is therefore urgent to improve the salt tolerance of Syn2973 for the future application of this promising chassis for green fuels and chemical production.

Acclimation to high external salinities involves the accumulation of compatible solutes and the active export of ions (Hagemann, 2011). Compatible solutes are a diverse class of low-molecular-weight compounds that accumulate at high intracellular concentrations in response to hyperosmotic or salt stress (Da Costa et al., 1998). Glucosylglycerol (GG) is a potent compatible solute consisting of glucose and glycerol linked by a glycosidic bond (Tan et al., 2016) and has a wide range of beneficial functions as a health food material, therapeutic agent, cosmetic ingredient, and enzyme stabilizer (Sawangwan et al., 2010; Luley-Goedl and Nidetzky, 2011). The marine cyanobacterium *Synechococcus* sp. PCC 7002 (hereafter Syn7002) and moderate halotolerant cyanobacterium *Synechocystis* sp. PCC 6803 (hereafter Syn6803) could accumulate GG to resist salt stress (Hagemann, 2011). The synthesis of GG has been elucidated in Syn6803, which depends strictly on ADP-glucose and consists of two steps involved in two enzymes [GG-phosphate-synthase (GGPS) and GG-phosphate-phosphatase (GGPP)] (Hagemann and Erdmann, 1994). In addition, the regulatory mechanisms involved in the synthesis of GG have mainly been investigated in *Synechocystis* (Kirsch et al., 2019). However, GG was not synthesized natively in Syn2973; instead, sucrose was synthesized as the only compatible solute under salt stress (Song et al., 2016). Heterologous synthesis of compatible solutes in salt-sensitive strains has been considered a key strategy for improving the salt tolerance of hosts (Waditee-Sirisattha et al., 2012; Singh et al., 2013). For example, the introduction of glycine methylation genes (*ApGSMT-DMT*) associated with glycine betaine synthesis successfully improved the salt tolerance of *Anabaena* sp. PCC 7120, and the growth of *Anabaena* sp. The PCC 7120-expressing *ApGSMT-DMT* gene cluster was improved by ∼70% under conditions with 0.12 M NaCl (Waditee-Sirisattha et al., 2012). It is thus interesting to explore the effect of heterologous GG synthesis on the salt tolerance of Syn2973. Notably, Syn2973 is known to undergo higher carbon flux into the sugar phosphate pathway and accumulates greater amounts of glycogen and sucrose (Abernathy et al., 2017; Ungerer et al., 2018a), which might also be beneficial for the heterologous synthesis of GG to improve salt tolerance.

**FIGURE 1:**
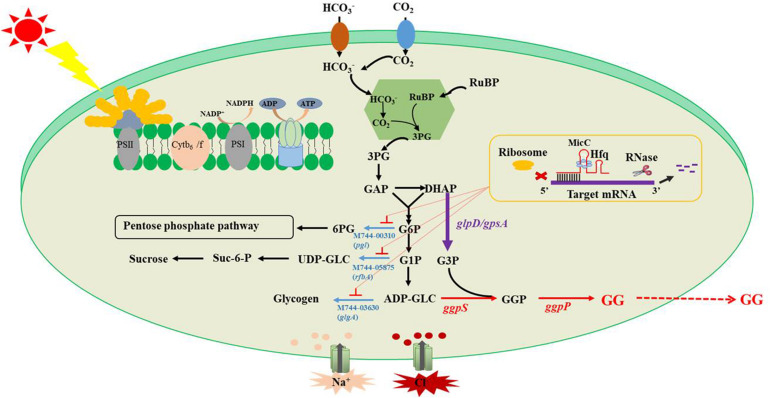
Schematic diagram of GG synthesis optimization.

In this study, multiple efforts were made to improve the salt tolerance of Syn2973 by achieving highly efficient biosynthesis of GG (Figure 1). Specifically, GG biosynthesis was first established in Syn2973 upon introducing genes encoding GgpS and GgpP. Second, the rate-limiting step in the process of converting DHAP to G3P was identified and strengthened by overexpressing genes from different sources. Third, redirection of carbon flux toward GG production by a small RNA (sRNA) regulatory tool was performed. Finally, the salt tolerance of different mutants was tested under different culture conditions. Together, these integrated efforts improved the salt tolerance of Syn2973 by 62% and represent the first attempt to increase salt stress tolerance by *de novo* biosynthesis of GG in Syn2973.

## Materials and Methods

### Bacterial Growth Conditions

The wild-type Syn2973 and engineered strains were grown on BG11 medium (pH 7.5) under a light intensity of approximately 200 μmol photons m^–2^ s^–1^ or 500 μmol photons m^–2^ s^–1^ in a shaking incubator at 130 rpm at 37°C (HNYC-202T, Honour, Tianjin, China) (Li et al., 2018). CO_2_ from the air was used as the only carbon source. Appropriate antibiotics, 20 μg/ml spectinomycin or chloromycetin (Solarbio, Beijing, China), were added to maintain the stability of the Syn2973 engineered strains. Inoculum and 0 M NaCl BG11 medium were mixed with 1 M NaCl BG11 medium and adjusted to a certain NaCl concentration. Cell optical density was measured by a spectrophotometer (UV-1750, Shimadzu, Kyoto, Japan) at 750 nm. *Escherichia coli* DH5α was grown on LB agar plates or in LB liquid medium in an incubator at 37°C or shaking incubator at 200 rpm supplemented with 50 μg/ml spectinomycin or 200 μg/ml ampicillin (Solarbio, Beijing, China).

For GG production, prior to production experiments, mutants were inoculated in BG11 medium containing appropriate antibiotics and grown photoautotrophically. For the production experiments, mutant cultures were incubated in fresh BG11 medium without antibiotics under different NaCl concretions at the initial OD_750_ of 0.1 and cultivated photoautotrophically.

### Construction of Strains and Plasmids

The strains and plasmids used in this study are listed in Table 1. Among them, *E. coli* DH5α was used for vector construction and amplification. For gene overexpression, the integrative vector pSI with a spectinomycin-resistant cassette and pSII with a chloromycetin-resistant cassette were used (Li et al., 2018). Primers for gene amplification are listed in Supplementary Table 1. All primers were synthesized by GENEWIZ Inc. (Suzhou, China). The target genes were amplified by Phanta Super-Fidelity DNA Polymerase (Vazyme Biotech, Nanjing, China), purified by a Cycle Pure Kit (Omega Bio-Tek, Norcross, GA, United States), and then phosphorylated by T4 Polynucleotide Kinase (Thermo Fisher Scientific Inc., CA, United States). Then, the gene products were ligated into pSI/pSII (linearized by PCR) by blunt end connection. All constructs were validated by colony PCR and confirmed by Sanger sequencing. The constructed plasmid was finally transferred into Syn2973 according to a method reported previously (Li et al., 2018). Briefly, *E. coli* HB101 harboring pRL443 and pRL623 and *E. coli* DH5α harboring the target plasmid were cultured overnight and then transferred into fresh LB medium with 50 μg/ml spectinomycin or 200 μg/ml ampicillin (Solarbio, Beijing, China) at a 1:50 ratio. When cells grew to exponential phase (OD_600_ ≈ 0.5), 2 ml of each *E. coli* strain was washed twice with fresh (LB) medium to remove all antibiotics, resuspended in 0.1 ml of LB medium, mixed together, and incubated for 30 min. One milliliter of the exponentially growing Syn2973 (OD_750_ ≈ 1) culture was centrifuged and resuspended in 0.2 ml of BG11 medium for each conjugation. The sample was then mixed with the *E. coli* suspension mentioned above and incubated for 30 min. The mixtures were spread on sterile filters (0.45 μm pore size) coated on BG11 agar plates. After incubation for 24 h at an intensity of approximately 100 μmol photons m^–2^ s^–1^, the filter was transferred onto new BG11 agar plates with 80 μg/ml spectinomycin or chloromycetin. Clones were observed after incubation at an intensity of approximately 200 μmol photons m^–2^ s^–1^ for approximately 5 days. All strains obtained were validated by colony PCR (Supplementary Figure 1) and Sanger sequencing.

**TABLE 1:** Strains and plasmids used in this study.

Strains/plasmids	Genotype or relevant features	References
**Plasmids**		
pSI	NSI:P*_*trc*_-mcs*-T*_*rbcL*_*; *spe*^*R*^	Li et al., 2018
pSII	NSII:P*_*cpc*__560_-mcs*-T*_*rbcL*_*; *cm*^*R*^	Li et al., 2018
pBA3031 M	pBA3031 M:P*psbA2 M-aslacZ2-micC*-T*rbcL*-Pcpc560-*hfq*-T*rbcL*; *spe*^*R*^	Sun et al., 2018
**Strains**		
M-pSI	NSI:P*_*trc*_-mcs*-T*_*rbcL*_*; *spe*^*R*^	
M-GgpPS	NSI:P*_*trc*_-sll0746*-P*_*trc*_-sll1566*-T*_*rbcL*_*; *spe*^*R*^	In this study
M-1085-GgpPS	NSI:P*_*cpc*__560_-sll1085*-P*_*trc*_-sll0746*-P*_*trc*_-sll1566*-T*_*rbcL*_*; *spe*^*R*^	In this study
M-1175-GgpPS	NSI:P*_*cpc*__560_-sll1755*-P*_*trc*_-sll0746*-P*_*trc*_-sll1566*-T*_*rbcL*_*; *spe*^*R*^	In this study
M-2522-GgpPS	NSI:P*_*cpc*__560_-Syn2522*-P*_*trc*_-sll0746*-P*_*trc*_-sll1566*-T*_*rbcL*_*; *spe*^*R*^	In this study
M-Glpd1-GgpPS	NSI:P*_*cpc*__560_-glpd1*-P*_*trc*_-sll0746*-P*_*trc*_-sll1566*-T*_*rbcL*_*; *spe*^*R*^	In this study
M-pSI-pSII	NSI:P*_*trc*_-mcs*-T*_*rbcL*_*; *spe*^*R*^; NSII:P*_*cpc*__560_-mcs*-T*_*rbcL*_*; *cm*^*R*^	In this study
M-2522-GgpPS-pSII	NSI:P*_*cpc*__560_-Syn2522*-P*_*trc*_-sll0746*-P*_*trc*_-sll1566*-T*_*rbcL*_*; *spe*^*R*^; NSII:P*_*cpc*__560_-mcs*-T*_*rbcL*_*; *cm*^*R*^	In this study
M-2522-GgpPS-dpgl	NSI:P*_*cpc*__560_-Syn2522*-P*_*trc*_-sll0746*-P*_*trc*_-sll1566*-T*_*rbcL*_*; *spe*^*R*^; NSII:P*psbA2 M-aspgl-micC*-T*rbcL*-P*_*cpc*__560_-hfq*-T*rbcL*; *cm*^*R*^	In this study
M-2522-GgpPS-drfbA	NSI:P*_*cpc*__560_-Syn2522*-P*_*trc*_-sll0746*-P*_*trc*_-sll1566*-T*_*rbcL*_*; *spe*^*R*^; NSII:P*psbA2 M-asrfbA-micC*-T*rbcL*-P*_*cpc*__560_-hfq*-T*rbcL*; *cm*^*R*^	In this study
M-2522-GgpPS-dglgA	NSI:P*_*cpc*__560_-Syn2522*-P*_*trc*_-sll0746*-P*_*trc*_-sll1566*-T*_*rbcL*_*; *spe*^*R*^; NSII:P*psbA2 M-asglgA-micC*-T*rbcL*-P*_*cpc*__560_-hfq*-T*rbcL*; *cm*^*R*^	In this study

### GG and Sucrose Extraction and Measurement

Based on the methods of a previous study (with minor modifications) (Tan et al., 2015), samples (1 OD) of mid-exponential cultures under salt stress were centrifuged at 12,000 rpm for 5 min. Harvested cells were suspended in the same volume (0.8 ml) of 80% ethanol, incubated at 65°C for 4 h, and centrifuged at 12,000 rpm for 5 min; 0.2 ml of supernatant solution was taken for analysis, and internal standard 13C-sorbitol with a final certain concentration of 62.5 mg/L was added. The solvents were removed using a vacuum concentration system (ZLS-1, Her-exi, Hunan, China). For GC-MS analysis, each sample was further derivatized in a two-step process as previously published (Cui et al., 2016). The derivatized samples were analyzed by GCMS using an Agilent 5975 MSD/7890 instrument (Agilent Corp., Santa Clara, CA, United States). The column was a HP-5MS (Restek, Bellefonte, PA, United States). The oven temperature was initially held at 80°C for 2 min and reached 280°C at 10°C per min, then held at 280°C for 5 min. The GG production per OD was calculated by internal standard method. The dry cell weight corresponded to OD_750 *nm*_ by the regression equation *y* = 0.9*x* + 4E–5 (*r*^2^ = 0.9998, *p* < 0.05), where *y* is the dry cell weight (mg) and *x* is the absorbance of the cell suspension at 750 nm.

### LC-MS-Based Targeted Metabolomics Analysis

Samples (8 ml) of mid-exponential cultures (48 h) at an OD_750_ of 1 ± 0.1 were rapidly harvested by centrifugation at 8000 × *g* for 8 min at 25°C (Eppendorf 5430R, Hamburg, Germany). The extraction of metabolites was carried out as previously published with slight modifications (Bennette et al., 2011; Wang et al., 2014). ^13^C3,^15^N-alanine (Cambridge Isotope Laboratories, Inc., Andover, MA, United States) was added as the internal standard to correct for variation due to sample extraction and injection. Briefly, approximately eight OD_750_ cells were added to 900 μl of the solution containing methanol/H_2_O (8:2, *v*/*v*) and then frozen-thawed three times. Samples were centrifuged at 15,000 × *g* for 5 min at 4°C. The supernatant was extracted, and the sediment was subjected to the above extraction process. The supernatants were mixed, and the solvents were removed using a vacuum concentration system (ZLS-1, Her-exi, Hunan, China). For LC-MS analysis, each dried sample was dissolved in 100 μl of purified water.

LC-MS analysis was conducted on an Agilent 1260 series binary HPLC system (Agilent Technologies, Santa Clara, CA, United States) using an XBridge Amide column (150 × 2.1 mm, 3.5 μm; Waters, Milford, MA, United States) coupled to an Agilent 6410 550 triple quadrupole mass analyzer equipped with an electrospray ionization source (ESI). Multiple reaction monitoring (MRM) mode was used for scanning according to Cui et al. (2020b). All of the peaks were integrated by Qualitative Analysis B.06.00 software and Xcalibur (version 2.1) (Niu et al., 2015). Sucrose was derivatized and analyzed on an Agilent 5975B/6890N GC-MS instrument (Agilent Technologies, Santa Clara, CA, United States) as previously described (Cui et al., 2016). The metabolite data were normalized to internal standards and cell numbers. Glycogen extraction and determination were performed according to the method described by Song et al. (2016).

### Quantitative RT-PCR Analysis

Total RNA extraction of samples was achieved through a miRNeasy Mini Kit (Qiagen, Valencia, CA, United States) following the protocols described by Sun et al. (2017). In addition, DNase treatment after RNA preparation to ensure degradation of copurified gDNA and 500 ng of total RNA were subjected to cDNA synthesis using a RevertAid First Strand cDNA Synthesis Kit following the manufacturer’s protocol (Thermo Fisher Scientific Inc., CA, United States). Then, 1 μl of each dilution was used as a template for the qRT-PCR described previously (Sun et al., 2018). Three technical replicates were performed for each condition. Data analysis was carried out using StepOnePlus analytical software (Applied Biosystems, CA, United States) and the 2^–ΔΔ^ ^CT^ method (Livak and Schmittgen, 2001).

### Statistical Analysis

In this study, each experiment was performed with three biological replicates. All data were reported as the means ± standard deviations. A statistical *t*-test model was applied for the comparative analysis, and a *p*-value of less than 0.05 was considered statistically significant.

## Results

### Improved Salt Tolerance of Syn2973 by Heterologous Expression of the GG Biosynthetic Pathway

**FIGURE 2:**
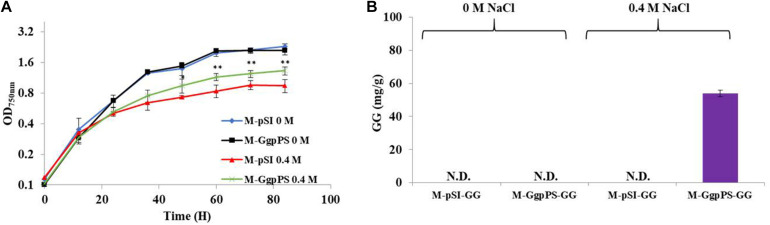
Growth curves and GG synthesis of strains M-pSI and M-GgpPS. **(A)** Growth curves of strains M-pSI and M-GgpPS under 0 M and 0.4 M NaCl; **(B)** GG synthesis of strain M-pSI and M-GgpPS under 0 M and 0.4 M NaCl. N.D., not detected.

To improve the salt tolerance of Syn2973, the synthetic pathway of the compatible solute GG was introduced into Syn2973. First, the *ggps* and *ggpp* genes were cloned from Syn6803 onto plasmid pSI with a strong P*trc* promoter. Then, the resulting plasmid was introduced into Syn2973, generating strain M-GgpPS. Compared with the control strain M-pSI, which only harbored the empty plasmid, the growth of strain M-GgpPS showed no difference under 0 M NaCl addition but was improved by approximately 24% under 0.4 M NaCl at a cultivation time point of 60 h (Figure 2A). In addition, a time course of GG production of strain M-GgpPS was determined under 0.4 M NaCl (Supplementary Figure 2), and the chromatogram analysis of GG is shown in Supplementary Figure 3. The results showed that GG production reached 51 mg/g under 0.4 M NaCl at a cultivation time point of 60 h. Meanwhile, no GG production was detected under normal conditions without salt stress (Figure 2B). Early studies showed that the GgpS protein is activated and catalyzes the synthesis of α-glucosylglycerol-phosphate in Syn6803 once under certain salt concentrations (Novak et al., 2011). Moreover, a recent study showed that NaCl stress was required to initiate αGG synthesis in *Corynebacterium glutamicum* cells, suggesting that the mechanism of osmosensing of GGPS by salt-dependent protein–nucleic acid interactions might be conserved even when the coding gene of the enzyme is transferred into a heterologous host (Roenneke et al., 2018). Consistently, our results showed that upon NaCl-induced stress, GgpS was activated in Syn2973, similar to Syn6803 (Tan et al., 2015), leading to the synthesis of GG. Furthermore, the synthesis of GG slightly enhanced the salt tolerance of Syn2973 (Figure 2A) under the tested condition. Next, it is necessary to improve GG synthesis to further enhance the salt tolerance of Syn2973.

### Screening the Rate-Limiting Steps of Precursor Supply for GG Synthesis

**FIGURE 3:**
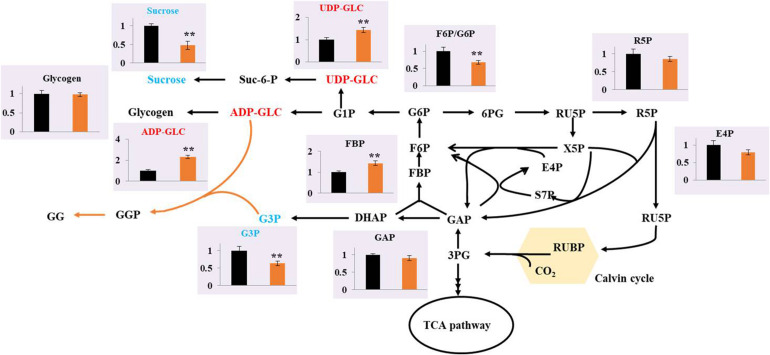
Comparison of the metabolites between the strains M-pSI (black) and M-GgpPS (orange) under 0.4 M NaCl. The *y*-axis is the ratio of the abundance of strain M-GgpPS to strain M-pSI under 0.4 M NaCl. The average value for strain M-pSI was set to 1. Data show the mean with error bars indicating the standard deviation calculated from three independent biological replicates.

Metabolomics, which involves the measurement of various cellular metabolites, is indispensable for understanding the cellular status, rate-limiting steps of metabolic flow, and productivity of metabolites. Metabolomics based on gas chromatography (GC), liquid chromatography (LC), and capillary chromatography with mass spectrometry (MS) have been performed to analyze cyanobacterial cells for the production of ethanol (Yoshikawa et al., 2017), succinic acid (Osanai et al., 2015), PHB (Arisaka et al., 2019), and others. To determine the possible regulatory mechanism relevant to GG biosynthesis in Syn2973, a better understanding of the intracellular metabolism in the GG-producing strain is required. Thus, the metabolites of strains M-pSI and M-GgpPS cultivated under 0.4 M NaCl conditions were extracted and subjected to LC-MS-based targeted metabolomics analysis. The constructed central metabolic pathways in Syn2973 are shown in Figure 3. Notably, the metabolomic results showed that the precursor metabolite of GG synthesis, G3P, was decreased by 26.02% in strain M-GgpPS compared with strain M-pSI, indicating that the synthesis of G3P might be a limiting step and could be further strengthened to improve GG biosynthesis. Interestingly, the metabolites that were involved in sucrose and glycogen metabolism, such as ADP-GLC and UDP-GLC, in strain M-GgpPS were increased by 2.31- and 1.44-fold compared with those of the control strain M-pSI, respectively. To further evaluate the effect of GG production on sucrose and glycogen metabolism, the contents of sucrose and glycogen were also measured, and the results showed that the content of sucrose was significantly decreased by approximately 50%, while no significant change in the glycogen content was observed in strain M-GgpPS. The glycogen synthase (encoded by gene *glgA*) incorporates glucose monomers into the growing 1,4-α-linked glucose polymer. UDP glucose pyrophosphorylase (encoded by gene *rfbA*) catalyzes the production of UDP-GLC from G1P and UTP. In addition, the sucrose phosphate synthase (SPS) and sucrose phosphophosphatase (SPP) encoded by gene *Syn0808* catalyzes the synthesis of sucrose from UDP-GLC and F6P. Previous studies have shown that this enzyme reaction is the limiting step of sucrose synthesis in cyanobacteria (Du et al., 2013; Lin et al., 2020). To further explore the effects of GG synthesis on sucrose and glycogen metabolism, qRT-PCR was performed to determine the mRNA abundance of three target genes (*glgA*, *rfbA*, and *Syn0808*) of strains M-pSI and M-GgpPS under salt stress. As illustrated in Supplementary Figure 4, the transcriptional levels of *glgA* and *rfbA* were increased by 39 and 62%, respectively, while the transcriptional level of *Syn0808* was decreased by 42% compared with that of strain M-pSI. In addition, the metabolites R5P and E4P associated with the pentose phosphate pathway were decreased by 13.8 and 20.2%, respectively. These results suggested that more carbon flux might be redirected though sucrose and GG metabolism in the GG-producing strain M-GgpPS, while decreased carbon flux was directed to the pentose phosphate pathway. We next sought to enhance GG synthesis in Syn2973 by improving G3P contents and regulating ADP-GLC flux toward GG biosynthesis and away from glycogen and sucrose biosynthesis.

### Enhancing GG Synthesis by Overexpressing the Glycerol-3-Phosphate Dehydrogenase Gene

**FIGURE 4:**
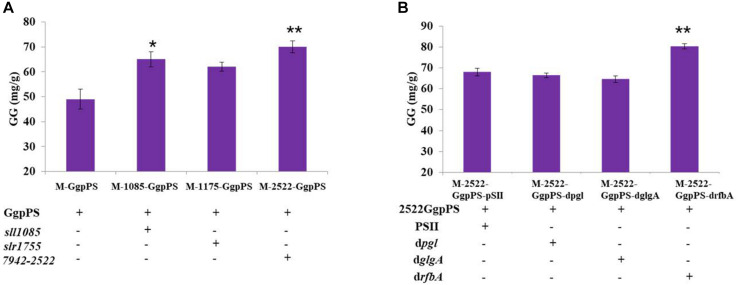
**(A)** GG synthesis of strains M-GgpPS, M-1085-GgpPS, M-1175-GgpPS, and M-2522-GgpPS; **(B)** GG synthesis of strains M-2522-GgpPS-pSII, M-2522-GgpPS-dpgl, M-2522-GgpPS-drfbA, and M-2522-GgpPS-dglgA. Statistical analysis was conducted as described in the text, as statistical significance indicated by **p < 0.01; *p < 0.05.

To further improve GG synthesis, heterologous genes that encoded glycerol-3-phosphate dehydrogenase (GlpD) were overexpressed in Syn2973 to enhance G3P supply and GG synthesis. In detail, four genes, *sll1085* encoding GlpD (glycerol-3-phosphate dehydrogenase) from Syn6803, *slr1755* encoding GpsA (NAD^+^-dependent glycerol-3-phosphate dehydrogenase) from Syn6803, Synpcc7942-2522 from Syn7942, and YDL022 W (*glpd1*) from *S. cerevisiae*, were cloned into the plasmid pSI-*ggpPS* with a strong P*cpc560* promoter and introduced into the wild-type Syn2973 strain. The strain M-Glpd1-GgpPS overexpressing *glpd1* could not survive under salt stress (data not shown), indicating that *glpd1* from *S. cerevisiae* might be toxic to Syn2973 under salt stress, although it had a positive effect on the G3P supply for lipid accumulation in Syn6803 (Wang et al., 2016). Then, the total GG production of strains M-1085-GgpPS, M-1175-GgpPS, and M-2522-GgpPS reached 65, 62, and 70 mg/g at a cultivation time point of 60 h, respectively, improving 32, 26, and 42% compared with strain M-GgpPS, respectively (Figure 4A).

### Knockdown of the Competing Pathway Using Hfq-MicC to Improve GG Production

The glycogen, sucrose, and pentose phosphate pathways are the major carbon competition pathways for GG synthesis (Figure 1). However, knockout of genes in essential pathways might cause severe growth inhibition. An sRNA regulatory tool that introduces an exogenous Hfq chaperone and MicC scaffold with a binding sequence (Hfq-MicC) was developed in Syn6803, achieving the accurate regulation of endogenous and exogenous genes (Sun et al., 2018). Recently, the utilization of the Hfq-MicC tool was also established and tested in Syn2973, suggesting that this tool was effective in Syn2973 and demonstrated the essential role of Hfq (Li et al., 2018). To drive more carbon flux from the competing pathways to GG biosynthesis, the sRNA regulatory tool above was employed, and the M744-00310-encoding gene *pgl* related to the pentose phosphate pathway, the M744-05875-encoding gene *rfbA* related to sucrose synthesis, and the M744-03630-encoding gene *glgA* related to glycogen synthesis were selected as target genes. The strains M-2522-GgpPS-dpgl, M-2522-GgpPS-dglgA, and M-2522-GgpPS-drfbA were constructed, targeting the *pgl*, *rfbA*, and *glgA* genes, respectively (Table 1). qRT-PCR assays were carried out to determine the mRNA abundances of three target genes in the mutant strains. As illustrated in Supplementary Figure 5, the transcriptional levels of *pgl*, *rfbA*, and *glgA* were decreased by 52, 45, and 38%, respectively, compared to those in the control strain M-2522-GgpPS-pSII. In addition, the GG production of strains M-2522-GgpPS-pSII, M-2522-GgpPS-dpgl, M-2522-GgpPS-dglgA, and M-2522-GgpPS-drfbA achieved 68, 66, 64, and 80 mg/g, respectively, at a cultivation time point of 60 h (Figure 4B). Compared with the control strain M-2522-GgpPS-pSII, GG synthesis in strains M-2522-GgpPS-dpgl and M-2522-GgpPS-dglgA was slightly decreased, while GG production in strain M-2522-GgpPS-drfbA was increased by 17%. The GG productivity was up to 1.3 mg/g/h, which represented an increase of 56.8% compared with the initial strain M-GgpPS. Together, these results demonstrated that among these three tested genes, downregulating the gene *rfbA* produced the best enhancement of GG production.

### Comparative Targeted Metabolomics Analysis of Strains M-2522-GgpPS-drfbA and M-pSI-pSII

**FIGURE 5:**
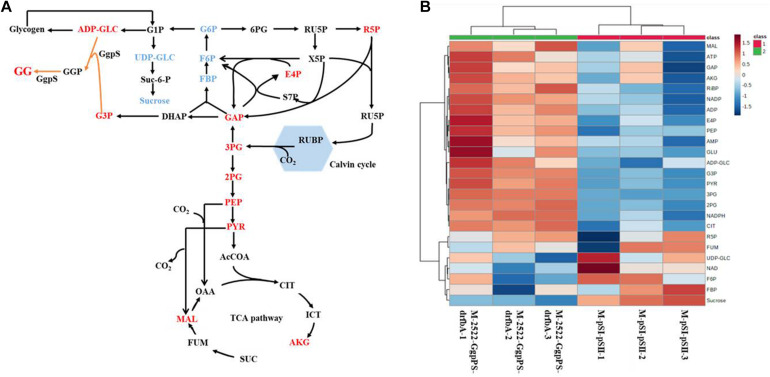
Targeted metabolomics analysis of strain M-2522-GgpPS-drfbA and M-pSI-pSII. **(A)** Comparison of the metabolites in central metabolic pathway between strain M-2522-GgpPS-drfbA and the control strain M-pSI-pSII, red: increased, blue: decreased; **(B)** heatmaps of metabolomics profiles in strains M-2522-GgpPS-drfbA and M-pSI-pSII. Each colored cell on the heatmap corresponds to a concentration value. The higher the concentration, the darker the color (red represents the increase, and blue represents the decrease).

To further explore the mechanisms relevant to high GG biosynthesis, the metabolites of strain M-2522-GgpPS-drfbA and the control strain M-pSI-pSII under 0.4 M NaCl and 200 μmol photons m^–2^ s^–1^ conditions were extracted and subjected to LCMS-based targeted metabolomics analysis. As shown in Figure 5, the G3P content in strain M-2522-GgpPS-drfbA was increased by threefold compared to the control strain M-pSI-pSII, suggesting that it was effective for G3P and GG accumulation by overexpressing the gene *glpD*. The UDP-GLC in strain M-2522-GgpPS-drfbA was decreased by 24%, while sucrose was further decreased by 60% compared with the control strain M-pSI-pSII, suggesting that more carbon flux might be redirected from sucrose metabolism to GG synthesis. The key metabolites of the gluconeogenic pathway in strain M-2522-GgpPS-drfbA, such as FBP and F6P, were decreased by 11 and 12%, respectively. However, GAP as the precursor to G3P was increased by 25% compared with the control strain M-pSI-pSII. In addition, the metabolites 3PG, PEP and PYR in the glycolytic pathway were increased by 6-, 1. 9-, and 2.4-fold compared with the control strain M-pSI-pSII, respectively, suggesting that more carbon might be redirected to the TCA cycle, which is necessary for biomass accumulation.

### GG Synthesis for the Salt Tolerance of Syn2973

**FIGURE 6:**
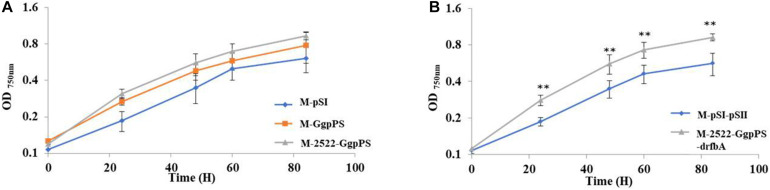
Growth curves of strains under 0.5 M NaCl and 200 μmol photons m^–2^ s^–1^ conditions. **(A)** Growth curves of strains M-pSI, M-GgpPS, and M-2522-GgpPS; **(B)** growth curves of strains M-2522-GgpPS-pSII and M-2522-GgpPS-drfbA. Statistical analysis was conducted as described in the text, as statistical significance indicated by ^∗∗^*p* < 0.01.

Glucosylglycerol, a compatible solute, plays an important role in moderate and halotolerant cyanobacterial strains with salt tolerance (Klahn and Hagemann, 2011). To test the salt tolerance of different GG-producing strains, growth curves of strains were measured under added 0.5 M NaCl. The results showed that the OD_750_ values of strains M-GgpPS and M-2522-GgpPS were increased by 27 and 52% compared with the control strain M-pSI at a cultivation time point of 60 h, respectively. The OD_750_ value of strain M-2522-GgpPS-drfbA was improved by 62% compared with that of the control strain M-pSI-pSII (Figure 6). These results indicated that the salt tolerance of Syn2973 was enhanced in association with the increase in GG synthesis in Syn2973.

In addition, the growth of strain M-2522-GgpPS-drfbA and control strain M-pSI-pSII was also measured under high light (500 μmol photons m^–2^ s^–1^) and salt stress conditions, and the growth of strain M-2522-GgpPS-drfbA was improved by threefold compared with the control strain M-pSI-pSII under high light and 0.4 M NaCl (Supplementary Figure 6). However, neither of the strains survived under high light and 0.5 M NaCl, suggesting that further improvement is still necessary for growth under high-light and high-salt conditions.

## Discussion

Syn2973 has a faster growth rate and is more tolerant to high temperature and high light, making it a good candidate chassis for autotrophic photosynthetic microbial cell factories. However, Syn2973 is sensitive to salt stress. In this study, the salt tolerance of Syn2973 was preliminarily improved by heterologously expressing *ggpP* and *ggpS*. Overexpressing the *glpD* gene and downregulating the *rfbA* gene further improved GG production and enhanced the salt tolerance of Syn2973. Under 0.5 M NaCl, the OD_750_ value of strain M-2522-GgpPS-drfbA was improved by 62% compared with that of the control strain M-pSI-pSII. A comparative metabolomic analysis between strains M-pSI-pSII and M-2522-GgpPS-drfbA showed that more carbon flux was redirected from ADP-GLC to GG synthesis. In addition, more carbon was directed to the TCA cycle for cell growth compared with the control strain M-pSI-pSII.

Glucosylglycerol synthesis was achieved in Syn2973 under salt stress conditions, and GG production reached 51 mg/g under salt stress in strain M-GgpPS. The GG production in wild-type *Synechocystis* was up to 100 mg/L (Tan et al., 2015), suggesting that the accumulation level of GG was relatively lower than that in *Synechocystis*. Reasons for the limited accumulation of GG in Syn2973 cells are not entirely clear but could be speculated, such as insufficient precursor supply. To confirm this hypothesis, targeted metabolomics analysis was conducted, and the results showed that the G3P content was decreased in strain M-GgpPS, suggesting that it was a limiting step for GG synthesis. G3P is a key precursor for triacylglycerol synthesis (Dulermo and Nicaud, 2011; Wang et al., 2016). A previous study showed that lipid production was enhanced by overexpressing the *glpD* gene (Wang et al., 2016). In addition, the production of 1,3-propanediol was increased by heterologous overexpression of the *glpD* gene (Liu et al., 2018). It is interesting to explore the relationship between GG synthesis and G3P supply. Moreover, ADP-GLC and UDP-GLC in strain M-GgpPS were increased, and pentose phosphate pathways, such as E4P and R5P, were decreased, suggesting that there might be more flux through the sucrose phosphate pathway due to the introduction of GG synthesis. The sucrose content decreased, which might be due to GG synthesis driving part of the flux. The qRT-PCR assay showed that expression of the *sps* gene was downregulated and that of *rfbA* was upregulated. The *sps* gene in Syn2973 was the limiting step for sucrose synthesis (Lin et al., 2020), suggesting that some carbon might accumulate in UDP-GLC. Cyanobacterial glycogen can be utilized as a sugar feedstock by microbes for ethanol fermentation (Aikawa et al., 2013) and has significant roles in tolerance to nitrogen, salt, or oxidative stress. Previous studies showed that sucrose and GG could be enhanced by blocking glycogen synthesis (Miao et al., 2003; Xu et al., 2013). The glycogen content did not show a significant change in strain M-GgpPS. It is necessary to enhance GG synthesis by improving G3P contents and directing ADP-GLC flux toward GG and away from glycogen and sucrose biosynthesis in future research.

By performing overexpression of *glpD* genes from different sources, we found that *glpD* from Syn7942 demonstrated the best effect in terms of improved GG synthesis, suggesting that it was effective for improving GG production by increasing the G3P supply. Surprisingly, GG production by strain M-2522-GgpPS-dglgA with *glgA* downregulation was decreased. A recent study showed that downregulation of glycogen has little effect on sucrose production (Lin et al., 2020). Another study suggested that reducing glycogen content could not increase sucrose content in Syn7942 (Ducat et al., 2012). Meanwhile, a previous study indicated a close link between the glycogen pool and compatible solute synthesis in Syn7942 and strongly suggested that intracellular glycogen serves, at least partially, as a carbon pool supporting sucrose synthesis rather than competing with sucrose synthesis (Qiao et al., 2018). A previous study also showed that overexpression of genes associated with glycogen synthesis, such as ADP-glucose pyrophosphorylase (GlgC), could enhance sucrose production (Lin et al., 2020). More recently, a study showed that trehalose production was increased by overexpressing *glgC* and isoamylase-type debranching enzyme (*glgX*) (associated with glycogen degradation) (Choi and Woo, 2020). It is thus worth further exploring how to increase glycogen metabolism for GG synthesis in the future. In addition, GG synthesis in strain M-2522-GgpPS-dpgl with downregulation of *pgl* was slightly decreased. The reason might be associated with the roles of the *pgl* gene in the pentose phosphate pathway, which was associated with CO_2_ fixation. The GG synthesis of strain M-2522-GgpPS-drfbA was increased by downregulating the *rfbA* gene, suggesting that more carbon flux was redirected from sucrose to GG synthesis.

To further decipher the possible mechanisms involved in GG synthesis and its regulation, targeted metabolomics analysis was also performed for strain M-2522-GgpPS-drfbA and the control strain M-pSI-pSII. The G3P in strain M-2522-GgpPS-drfbA was increased, suggesting that overexpressing *glpD* was effective for G3P accumulation. In addition, sucrose as a compatible solute in strain M-2522-GgpPS-drfbA was further decreased, which was closely related to the limited improvement of salt tolerance. The key metabolites of the gluconeogenic pathway in strain M-2522-GgpPS-drfbA, such as FBP, F6P, and GAP, were decreased. A previous study showed that fructose 1,6-bisphosphate aldolase (FBA) was associated with glycolysis, gluconeogenesis, and the Calvin cycle (Zgiby et al., 2000), which is known to be associated with salt stress (Patipong et al., 2019). It is interesting to check whether improving FBA activity could further redirect more carbon flux to the sugar synthesis pathway to further improve GG production. The metabolites 3PG, PEP, and PYR in the glycolytic pathway were increased compared with the control strain M-pSI-pSII, which was consistent with the better growth phenotype of strain M-2522-GgpPS-drfbA. It is expected that more carbon flow can be driven into the TCA cycle to maintain cell growth in the early stage, and more carbon flow can be redirected to the gluconeogenic pathway to promote GG synthesis by induced sRNA interference in the later stage.

Glucosylglycerol represents the primary compatible solute for salt resistance in many marine cyanobacteria (Kirsch et al., 2019). To validate whether the GG accumulation level was associated with the salt tolerance of Syn2973, different mutant strains were chosen to evaluate their growth under salt stress. The results suggested that the salt tolerance of mutants increased with increasing GG production ability, indicating that GG production was beneficial for Syn2973 under salt stress. Together, these results indicated that the heterologous synthetic compatible solutes could function as compatible solutes for resisting salt stress. In addition, the strain M-2522-GgpPS-drfbA and control strain M-pSI-pSII were tested under high-light culture conditions. The growth of strain M-2522-GgpPS-drfbA was far better than that of control strain M-pSI-pSII under high light and 0.4 M NaCl; however, neither of the strains survived under conditions of high light and 0.5 M NaCl, suggesting that high light could inhibit the growth of Syn2973 under salt stress. It is speculated that several mechanisms might be involved: (*i*) excess light energy could break the balance between energy supply and consumption of cyanobacteria, leading to the intracellular accumulation of reactive oxygen species (ROS) (Muramatsu and Hihara, 2012); meanwhile, high salt stress could also induce ROS accumulation (Swapnil et al., 2017). The accumulated ROS further disrupted key cellular components, seriously restricting cell viability; (*ii*) salt stress inhibited photosystems II and I in cyanobacteria (Allakhverdiev and Murata, 2008), which could interfere with photosynthesis under high light; and (*?*) a previous study showed that energy metabolites such as ATP and NADPH might be necessary contributors to the rapid growth of Syn2973 under high-light conditions (Ungerer et al., 2018b). However, energy metabolites are also directly associated with Na^+^ excretion from the cytoplasm, such as Na^+^/H^+^ antiporters that utilize the energy of the transmembrane proton gradient for Na^+^ exclusion (Cui et al., 2020a). Therefore, energy metabolites might be urgent for cell growth under high-light and high-salt-stress conditions. In this study, we preliminarily tested the heterologous synthesis of GG and its effect on salt tolerance of Syn2973 under low CO_2_ concentration from the air, the same condition for both engineered strain and wild-type, which could tell the preliminary difference. It also worth evaluating the detailed performance of the engineered strains and wild-type employing other conditions or methods, such as under carbon-sufficient cultivation condition, or a spot assay on agar plates for salt tolerance monitoring in future research. Together, the study provides important engineering strategies to further improve salt tolerance and GG production of Syn2973 in the near future, and further work is still necessary to engineer Syn2973 to reach high salt and high light tolerance for future cultivation applications.

## Data Availability Statement

The datasets presented in this study can be found in online repositories. The names of the repository/repositories and accession number(s) can be found in the article/Supplementary Material.

## Author Contributions

LC and WZ designed the research. JC and TS performed the major experiments and wrote the draft manuscript. JC, TS, LC, and WZ analyzed the data and drafted and revised the manuscript. All authors contributed to the article and approved the submitted version.

## Conflict of Interest

The authors declare that the research was conducted in the absence of any commercial or financial relationships that could be construed as a potential conflict of interest.

## Supplementary Material

Supplementary Figure 1 Identification of correct strains by colony PCR. **(A)** Amplification of the fragment *Ptrc-sll0746-Ptrc-sll1566-TrbcL* from strains M-pSI (control strain) and M-GgpPS; **(B)** amplification of the fragment P*_*cpc560*_-Syn2522*-P*_*trc*_-sll0746*-P*_*trc*_-sll1566*-T*_*rbcL*_* from strains M-pSI and M-2522-GgpPS; **(C)** amplification of the fragment *asrfbA-micC-TrbcL-Pcpc560-hfq-TrbcL* from strains M-pSI-pSII (control strain) and M-2522-GgpPS-drfbA. M, marker.

Supplementary Figure 2 GG production at different cultivation points of strain M-GgpPS.

Supplementary Figure 3 Chromatograms of GG, sucrose and internal standard 13C-sorbitol. **(A)** Chromatogram of the derivatized 13C-sorbitol and GG standard; **(B)** chromatogram of control strain M-pSI; **(C)** chromatogram of strain M-GgpPS; **(D)** mass spectrometry of GG.

Supplementary Figure 4 Transcript level measurements of *glgA*, *rfbA*, and *Syn0808* in strains M-GgpPS (orange) and M-pSI (black) using qRT-PCR.

Supplementary Figure 5 Transcript level measurements of *pgl*, *glgA*, and *rfbA* in strains M-2522-GgpPS-dpgl, M-2522-GgpPS-dglgA, and M-2522-GgpPS-drfbA (orange) and the control strain M-2522-GgpPS-pSII (blue) using qRT-PCR.

Supplementary Figure 6 Growth curves of strains under salt and high light (500 μmol photons m^–2^ s^–1^) stress conditions.

Supplementary Table 1 Primers used in this study.

Supplementary Table 2 The raw metabolomics data of the heatmap.

## Abbreviations

AcCoA: acetyl-CoA
AKG: a-ketoglutarate
ADP-GLC: adenosine diphosphoglucose
ATP: adenosine triphosphate
CIT: citrate
D: day
DHAP: dihydroxyacetone phosphate
E4P: erythrose-4-phosphate
F6P: fructose-6-phosphate
FBP: fructose 1,6-diphosphate
G6P: glucose-6-phosphate
G1P: glucose 1-phosphate
G3P: glycerol-3-phosphate
GAP: glyceraldehyde 3-phosphate
3-PG: glycerate 3-phosphate
2-PG: glycerate-2-phosphate
GC-MS: gas chromatography mass spectrometry
h: hour
ICT: isocitrate
LC-MS: liquid chromatography mass spectrometry
MAL: malate
NADH: nitcotinamide adenine dinucleotide (reduced)
OAA: oxaloacetate
6PG: 6-phospho-gluconate
PYR: pyruvate
PEP: phosphoenolpyruvate
Ru5P: ribulose 5-phosphate
RUBP: ribulose 1,5-diphosphate
R5P: ribose 5-phosphate
S7P: seduheptulose-7-phosphate
SUC: succinate
Suc-6-P: sucrose 6-phosphate
UDP-GLC: uridine diphosphate glucose
UTP: uridine-5 ′ (1-thiotriphosphate)
X5P: xylulose 5-phosphate.

## References

[B1] Abernathy M. H. Yu J. Ma F. Liberton M. Ungerer J. Hollinshead W. D. (2017). Deciphering cyanobacterial phenotypes for fast photoautotrophic growth via isotopically nonstationary metabolic flux analysis. *Biotechnol. Biofuels* 10:273. 10.1186/s13068-017-0958-y PMC5691832 29177008

[B2] Aikawa S. Joseph A. Yamada R. Izumi Y. Yamagishi T. Matsuda F. (2013). Direct conversion of *Spirulina* to ethanol without pretreatment or enzymatic hydrolysis processes. *Energy Environ. Sci.* 6 1844–1849. 10.1039/C3EE40305J

[B3] Allakhverdiev S. I. Murata N. (2008). Salt stress inhibits photosystems II and I in cyanobacteria. *Photosynth. Res.* 98 529–539. 10.1007/s11120-008-9334-x 18670904

[B4] Arisaka S. Terahara N. Oikawa A. Osanai T. (2019). Increased polyhydroxybutyrate levels by *ntcA* overexpression in *Synechocystis* sp. PCC 6803. *Algal Res.* 41:101565. 10.1016/j.algal.2019.101565

[B5] Bennette N. B. Eng J. F. Dismukes G. C. (2011). An LC-MS-based chemical and analytical method for targeted metabolite quantification in the model cyanobacterium *Synechococcus* sp. PCC 7002. *Anal. Chem.* 83 3808–3816. 10.1021/ac200108a 21466217

[B6] Choi S. Y. Woo H. M. (2020). CRISPRi-dCas12a: a dCas12a-mediated CRISPR interference for repression of multiple genes and metabolic engineering in cyanobacteria. *ACS Synthet. Biol.* 9 2351–2361. 10.1021/acssynbio.0c00091 32379967

[B7] Cui J. Sun T. Chen L. Zhang W. (2020a). Engineering salt tolerance of photosynthetic cyanobacteria for seawater utilization. *Biotechnol. Adv.* 43:107578. 10.1016/j.biotechadv.2020.107578 32553809

[B8] Cui J. Sun T. Li S. Xie Y. Song X. Wang F. (2020b). Improved salt tolerance and metabolomics analysis of *Synechococcus elongatus* UTEX 2973 by Overexpressing Mrp Antiporters. *Front. Bioeng. Biotechnol.* 8:500. 10.3389/fbioe.2020.00500 32528943 PMC7264159

[B9] Cui J. Y. Good N. M. Hu B. Yang J. Wang Q. W. Sadilek M. (2016). Metabolomics revealed an association of metabolite changes and defective growth in *Methylobacterium extorquens* AM1 overexpressing *ecm* during growth on methanol. *PLoS One* 11:e0154043. 10.1371/journal.pone.0154043 27116459 PMC4846091

[B10] Da Costa M. S. Santos H. Galinski E. A. (1998). “An overview of the role and diversity of compatible solutes in *Bacteria* and *Archaea*,” in *Biotechnology of Extremophiles*, ed. Antranikian G. (Berlin: Springer), 117–153. 10.1007/BFb0102291 9670799

[B11] Du W. Liang F. Duan Y. Tan X. Lu X. (2013). Exploring the photosynthetic production capacity of sucrose by cyanobacteria. *Metab. Eng.* 19 17–25. 10.1016/j.ymben.2013.05.001 23721859

[B12] Ducat D. C. Avelar-Rivas J. A. Way J. C. Silver P. A. (2012). Rerouting carbon flux to enhance photosynthetic productivity. *Appl. Environ. Microbiol.* 78 2660–2668. 10.1128/aem.07901-11 22307292 PMC3318813

[B13] Dulermo T. Nicaud J. M. (2011). Involvement of the G3P shuttle and beta-oxidation pathway in the control of TAG synthesis and lipid accumulation in *Yarrowia lipolytica*. *Metab. Eng.* 13 482–491. 10.1016/j.ymben.2011.05.002 21620992

[B14] Gao X. Gao F. Liu D. Zhang H. Nie X. Yang C. (2016a). Engineering the methylerythritol phosphate pathway in cyanobacteria for photosynthetic isoprene production from CO_2_. *Energy Environ. Sci.* 9 1400–1411. 10.1039/c5ee03102h

[B15] Gao X. Sun T. Pei G. Chen L. Zhang W. (2016b). Cyanobacterial chassis engineering for enhancing production of biofuels and chemicals. *Appl. Microbiol. Biotechnol.* 100 3401–3413. 10.1007/s00253-016-7374-2 26883347

[B16] Gao Z. Zhao H. Li Z. Tan X. Lu X. (2012). Photosynthetic production of ethanol from carbon dioxide in genetically engineered cyanobacteria. *Energy Environ. Sci.* 5 9857–9865. 10.1039/c2ee22675h

[B17] Hagemann M. (2011). Molecular biology of cyanobacterial salt acclimation. *FEMS Microbiol. Rev.* 35 87–123. 10.1111/j.1574-6976.2010.00234.x 20618868

[B18] Hagemann M. Erdmann N. (1994). Activation and pathway of glucosylglycerol synthesis in the cyanobacterium *Synechocystis* sp. PCC 6803. *Microbiology* 140 1427–1431. 10.1099/00221287-140-6-1427

[B19] Herskowitz I. (1988). Life cycle of the budding yeast *Saccharomyces cerevisiae*. *Microbiol. Rev.* 52 536–553. 10.1128/mr.52.4.536-553.1988 3070323 PMC373162

[B20] Kirsch F. Klahn S. Hagemann M. (2019). Salt-regulated accumulation of the compatible solutes sucrose and glucosylglycerol in cyanobacteria and its biotechnological potential. *Front. Microbiol.* 10:2139. 10.3389/fmicb.2019.02139 31572343 PMC6753628

[B21] Klahn S. Hagemann M. (2011). Compatible solute biosynthesis in cyanobacteria. *Environ. Microbiol.* 13 551–562. 10.1111/j.1462-2920.2010.02366.x 21054739

[B22] Li S. Sun T. Xu C. Chen L. Zhang W. (2018). Development and optimization of genetic toolboxes for a fast-growing cyanobacterium *Synechococcus elongatus* UTEX 2973. *Metab. Eng.* 48 163–174. 10.1016/j.ymben.2018.06.002 29883802

[B23] Lin P. C. Zhang F. Pakrasi H. B. (2020). Enhanced production of sucrose in the fast-growing cyanobacterium *Synechococcus elongatus* UTEX 2973. *Sci. Rep.* 10:390. 10.1038/s41598-019-57319-5 PMC6962321 31942010

[B24] Liu H. Ni J. Xu P. Tao F. (2018). Enhancing light-driven 1,3-propanediol production by using natural compartmentalization of differentiated cells. *ACS Synth. Biol.* 7 2436–2446. 10.1021/acssynbio.8b00239 30234972

[B25] Livak K. J. Schmittgen T. D. (2001). Analysis of relative gene expression data using real-time quantitative PCR and the 2^–Δ^ ^Δ^ ^*CT*^ method. *Methods* 25 402–408. 10.1006/meth.2001.1262 11846609

[B26] Luan G. Lu X. (2018). Tailoring cyanobacterial cell factory for improved industrial properties. *Biotechnol. Adv.* 36 430–442. 10.1016/j.biotechadv.2018.01.005 29331411

[B27] Luley-Goedl C. Nidetzky B. (2011). Glycosides as compatible solutes: biosynthesis and applications. *Nat. Prod. Rep.* 28 875–896. 10.1039/c0np00067a 21390397

[B28] Matson M. M. Atsumi S. (2018). Photomixotrophic chemical production in cyanobacteria. *Curr. Opin. Biotechnol.* 50 65–71. 10.1016/j.copbio.2017.11.008 29179151

[B29] Miao X. Wu Q. Wu G. Zhao N. (2003). Sucrose accumulation in salt-stressed cells of *agp* gene deletion-mutant in cyanobacterium *Synechocystis* sp. PCC 6803. *FEMS Microbiol. Lett.* 218 71–77. 10.1111/j.1574-6968.2003.tb11500.x 12583900

[B30] Mueller T. J. Ungerer J. L. Pakrasi H. B. Maranas C. D. (2017). Identifying the metabolic differences of a fast-growth phenotype in *Synechococcus* UTEX 2973. *Sci. Rep.* 7:41569. 10.1038/srep41569 PMC5282492 28139686

[B31] Muramatsu M. Hihara Y. (2012). Acclimation to high-light conditions in cyanobacteria: from gene expression to physiological responses. *J. Plant Res.* 125 11–39. 10.1007/s10265-011-0454-6 22006212

[B32] Niu X. Zhang X. Yu X. Su Y. Chen L. Zhang W. (2015). Optimization and application of targeted LC-MS metabolomic analyses in photosynthetic cyanobacteria. *Chin. J. Biotechnol.* 31 577–590. 26380414

[B33] Novak J. F. Stirnberg M. Roenneke B. Marin K. (2011). A novel mechanism of osmosensing, a salt-dependent protein-nucleic acid interaction in the cyanobacterium *Synechocystis* Species PCC 6803. *J. Biol. Chem.* 286 3235–3241. 10.1074/jbc.m110.157032 21123179 PMC3030328

[B34] Osanai T. Shirai T. Iijima H. Nakaya Y. Okamoto M. Kondo A. (2015). Genetic manipulation of a metabolic enzyme and a transcriptional regulator increasing succinate excretion from unicellular cyanobacterium. *Front. Microbiol.* 6:1064. 10.3389/fmicb.2015.01064 26500619 PMC4594341

[B35] Patipong T. Ngoennet S. Honda M. Hibino T. Waditee-Sirisattha R. Kageyama H. (2019). A class I fructose-1,6-bisphosphate aldolase is associated with salt stress tolerance in a halotolerant cyanobacterium *Halothece* sp. PCC 7418. *Arch. Biochem. Biophys.* 672:108059. 10.1016/j.abb.2019.07.024 31356779

[B36] Qiao C. Duan Y. Zhang M. Hagemann M. Luo Q. Lu X. (2018). Effects of reduced and enhanced glycogen pools on salt-induced sucrose production in a sucrose-secreting strain of *Synechococcus elongatus* PCC 7942. *Appl. Environ. Microbiol.* 84:e002023-17. 10.1128/AEM.02023-17 PMC5752869 29101204

[B37] Roenneke B. Rosenfeldt N. Derya S. M. Novak J. F. Marin K. Kramer R. (2018). Production of the compatible solute alpha-D-glucosylglycerol by metabolically engineered *Corynebacterium glutamicum*. *Microb. Cell Fact* 17:94. 10.1186/s12934-018-0939-2 PMC6004087 29908566

[B38] Sawangwan T. Goedl C. Nidetzky B. (2010). Glucosylglycerol and glucosylglycerate as enzyme stabilizers. *Biotechnol. J.* 5 187–191. 10.1002/biot.200900197 19946880

[B39] Silkina A. Kultschar B. Llewellyn C. A. (2019). Far-red light acclimation for improved mass cultivation of cyanobacteria. *Metabolites* 9:170. 10.3390/metabo9080170 31430925 PMC6724174

[B40] Singh M. Sharma N. K. Prasad S. B. Yadav S. S. Narayan G. Rai A. K. (2013). The freshwater cyanobacterium *Anabaena doliolum* transformed with *ApGSMT-DMT* exhibited enhanced salt tolerance and protection to nitrogenase activity, but became halophilic. *Microbiology* 159 641–648. 10.1099/mic.0.065078-0 23329680

[B41] Song K. Tan X. Liang Y. Lu X. (2016). The potential of *Synechococcus elongatus* UTEX 2973 for sugar feedstock production. *Appl. Microbiol. Biotechnol.* 100 7865–7875. 10.1007/s00253-016-7510-z 27079574

[B42] Sun T. Li S. Song X. Pei G. Diao J. Cui J. (2018). Re-direction of carbon flux to key precursor malonyl-CoA via artificial small RNAs in photosynthetic *Synechocystis* sp. PCC 6803. *Biotechnol. Biofuels* 11:26. 10.1186/s13068-018-1032-0 PMC5798194 29441124

[B43] Sun T. Pei G. Wang J. Chen L. Zhang W. (2017). A novel small RNA CoaR regulates coenzyme A biosynthesis and tolerance of *Synechocystis* sp. PCC6803 to 1-butanol possibly via promoter-directed transcriptional silencing. *Biotechnol. Biofuels* 10:42. 10.1186/s13068-017-0727-y PMC5319066 28239414

[B44] Swapnil P. Yadav A. K. Srivastav S. Sharma N. K. Srikrishna S. Rai A. K. (2017). Biphasic ROS accumulation and programmed cell death in a cyanobacterium exposed to salinity (NaCl and Na_2_SO_4_). *Algal Res.* 23 88–95. 10.1016/j.algal.2017.01.014

[B45] Tan X. Du W. Lu X. (2015). Photosynthetic and extracellular production of glucosylglycerol by genetically engineered and gel-encapsulated cyanobacteria. *Appl. Microbiol. Biotechnol.* 99 2147–2154. 10.1007/s00253-014-6273-7 25503504

[B46] Tan X. Luo Q. Lu X. (2016). Biosynthesis, biotechnological production, and applications of glucosylglycerols. *Appl. Microbiol. Biotechnol.* 100 6131–6139. 10.1007/s00253-016-7608-3 27225470

[B47] Ungerer J. Lin P.-C. Chen H.-Y. Pakrasi H. B. (2018a). Adjustments to photosystem stoichiometry and electron transfer proteins are key to the remarkably fast growth of the cyanobacterium *Synechococcus elongatus* UTEX 2973. *mBio* 9:e02327-17. 10.1128/mBio.02327-17 PMC5801466 29437923

[B48] Ungerer J. Wendt K. E. Hendry J. I. Maranas C. D. Pakrasi H. B. (2018b). Comparative genomics reveals the molecular determinants of rapid growth of the cyanobacterium *Synechococcus elongatus* UTEX 2973. *Proc. Natl. Acad. Sci. U.S.A.* 115 E11761–E11770. 30409802 10.1073/pnas.1814912115 PMC6294925

[B49] Waditee-Sirisattha R. Singh M. Kageyama H. Sittipol D. Rai A. Takabe T. (2012). *Anabaena* sp. PCC7120 transformed with glycine methylation genes from *Aphanothece halophytica* synthesized glycine betaine showing increased tolerance to salt. *Arch. Microbiol.* 194 909–914. 10.1007/s00203-012-0824-z 22707090

[B50] Wang X. Xiong X. Sa N. Roje S. Chen S. (2016). Metabolic engineering of enhanced glycerol-3-phosphate synthesis to increase lipid production in *Synechocystis* sp. PCC 6803. *Appl. Microbiol. Biotechnol.* 100 6091–6101. 10.1007/s00253-016-7521-9 27154348

[B51] Wang J. Zhang X. Shi M. Gao L. Niu X. Te R. (2014). Metabolomic analysis of the salt-sensitive mutants reveals changes in amino acid and fatty acid composition important to long-term salt stress in *Synechocystis* sp. PCC 6803. *Funct. Integr. Genomics* 14 431–440. 10.1007/s10142-014-0370-7 24643737

[B52] Xu Y. Guerra L. T. Li Z. Ludwig M. Dismukes G. C. Bryant D. A. (2013). Altered carbohydrate metabolism in glycogen synthase mutants of *Synechococcus* sp. strain PCC 7002: Cell factories for soluble sugars. *Metab. Eng.* 16 56–67. 10.1016/j.ymben.2012.12.002 23262095

[B53] Yoshikawa K. Toya Y. Shimizu H. (2017). Metabolic engineering of *Synechocystis* sp. PCC 6803 for enhanced ethanol production based on flux balance analysis. *Bioprocess. Biosyst. Eng.* 40 791–796. 10.1007/s00449-017-1744-8 28258322

[B54] Yu J. Liberton M. Cliften P. F. Head R. D. Jacobs J. M. Smith R. D. (2015). *Synechococcus elongatus* UTEX 2973, a fast growing cyanobacterial chassis for biosynthesis using light and CO_2_. *Sci. Rep.* 5:8132. 10.1038/srep08132 PMC5389031 25633131

[B55] Zgiby S. M. Thomson G. J. Qamar S. Berry A. (2000). Exploring substrate binding and discrimination in fructose1,6-bisphosphate and tagatose 1,6-bisphosphate aldolases. *Eur. J. Biochem.* 267 1858–1868. 10.1046/j.1432-1327.2000.01191.x 10712619

