# Supplementary figures and images for: Salt-Tolerant Synechococcus elongatus UTEX 2973 Obtained via Engineering of Heterologous Synthesis of Compatible Solute Glucosylglycerol
Source: Front Microbiol. 2021 May 18;12:650217. doi: 10.3389/fmicb.2021.650217 (PMC8168540; doi:10.3389/fmicb.2021.650217)

## Slide 1
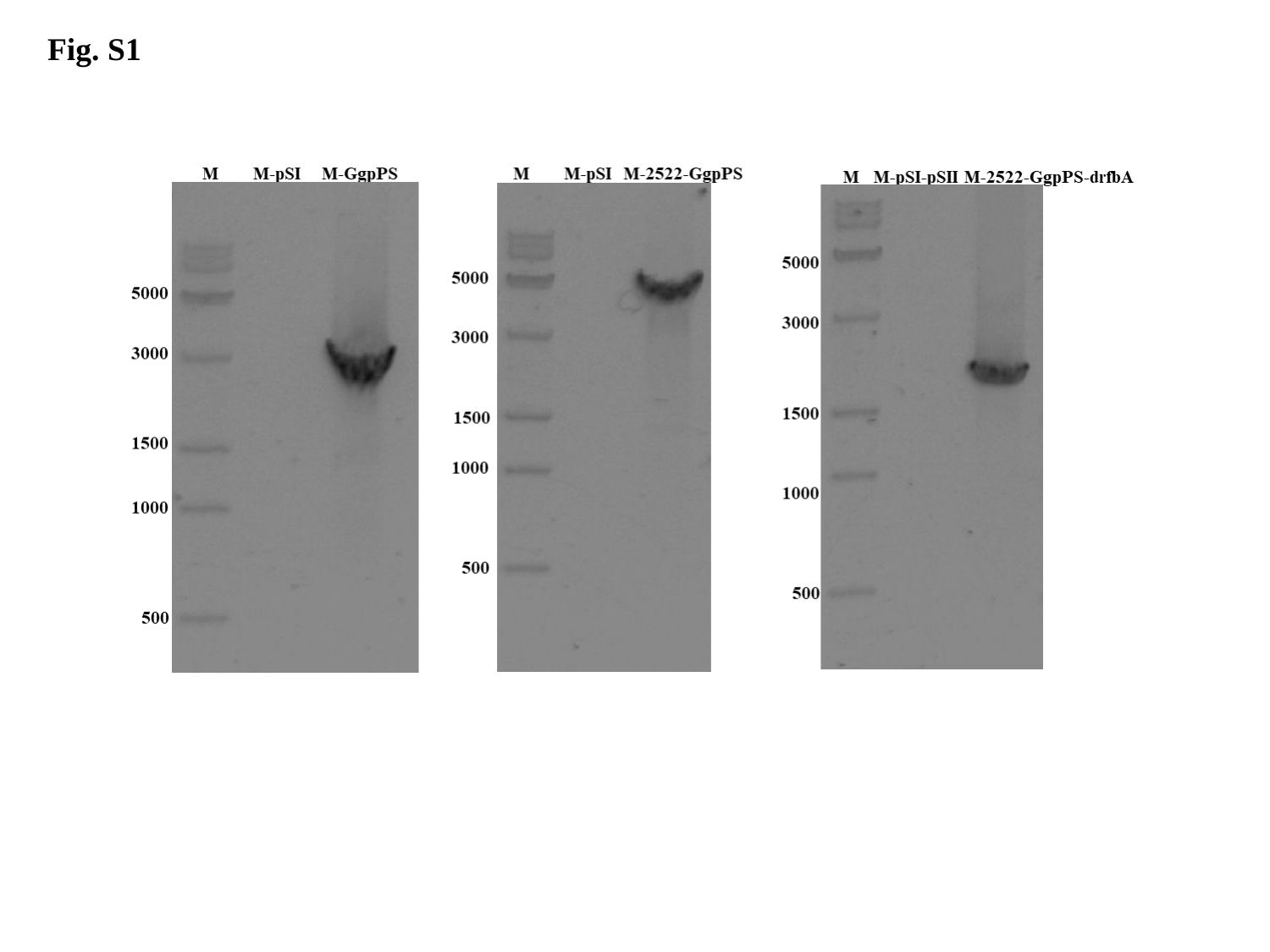

Fig. S1

## Slide 2
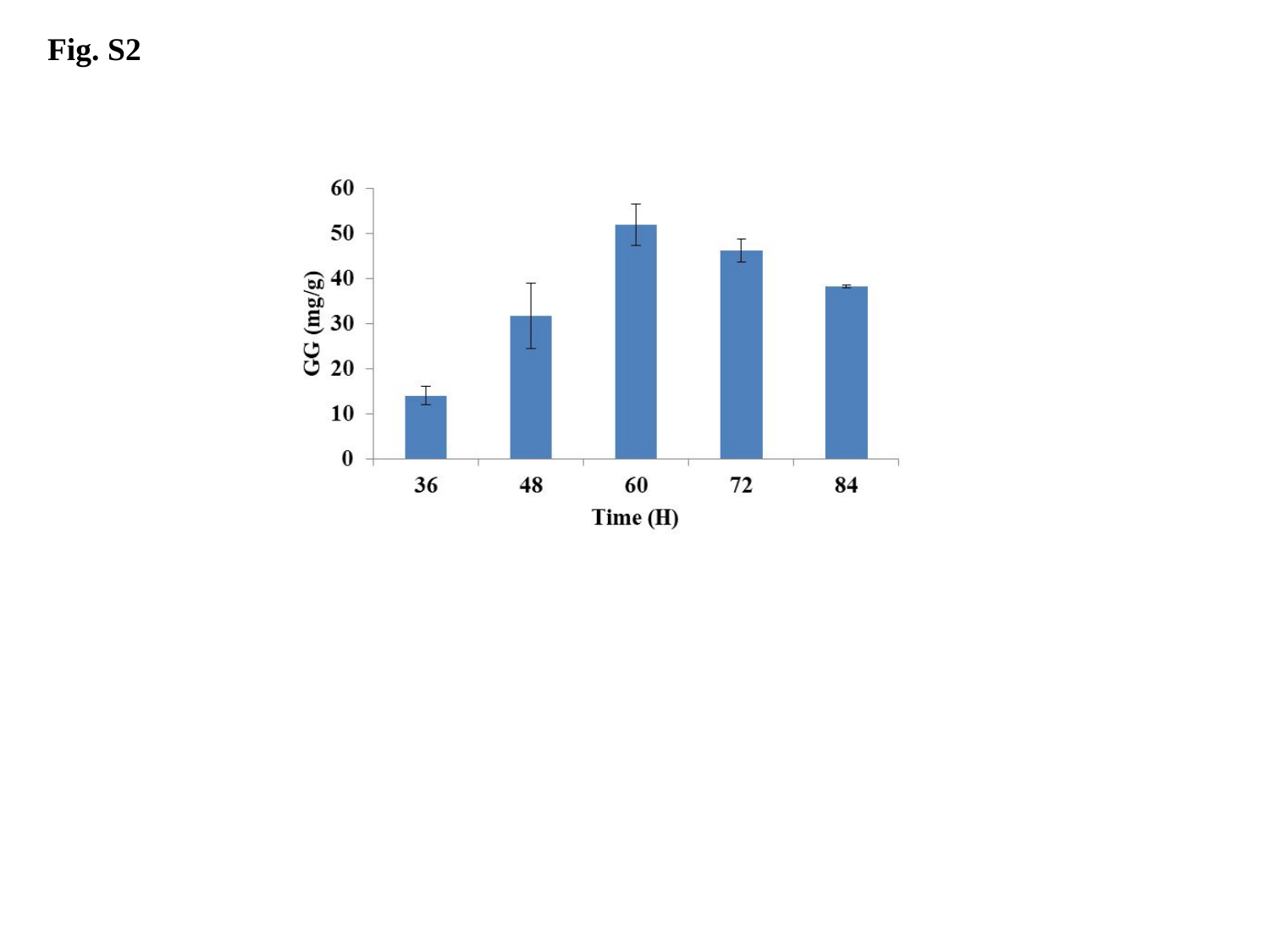

Fig. S2

## Slide 3
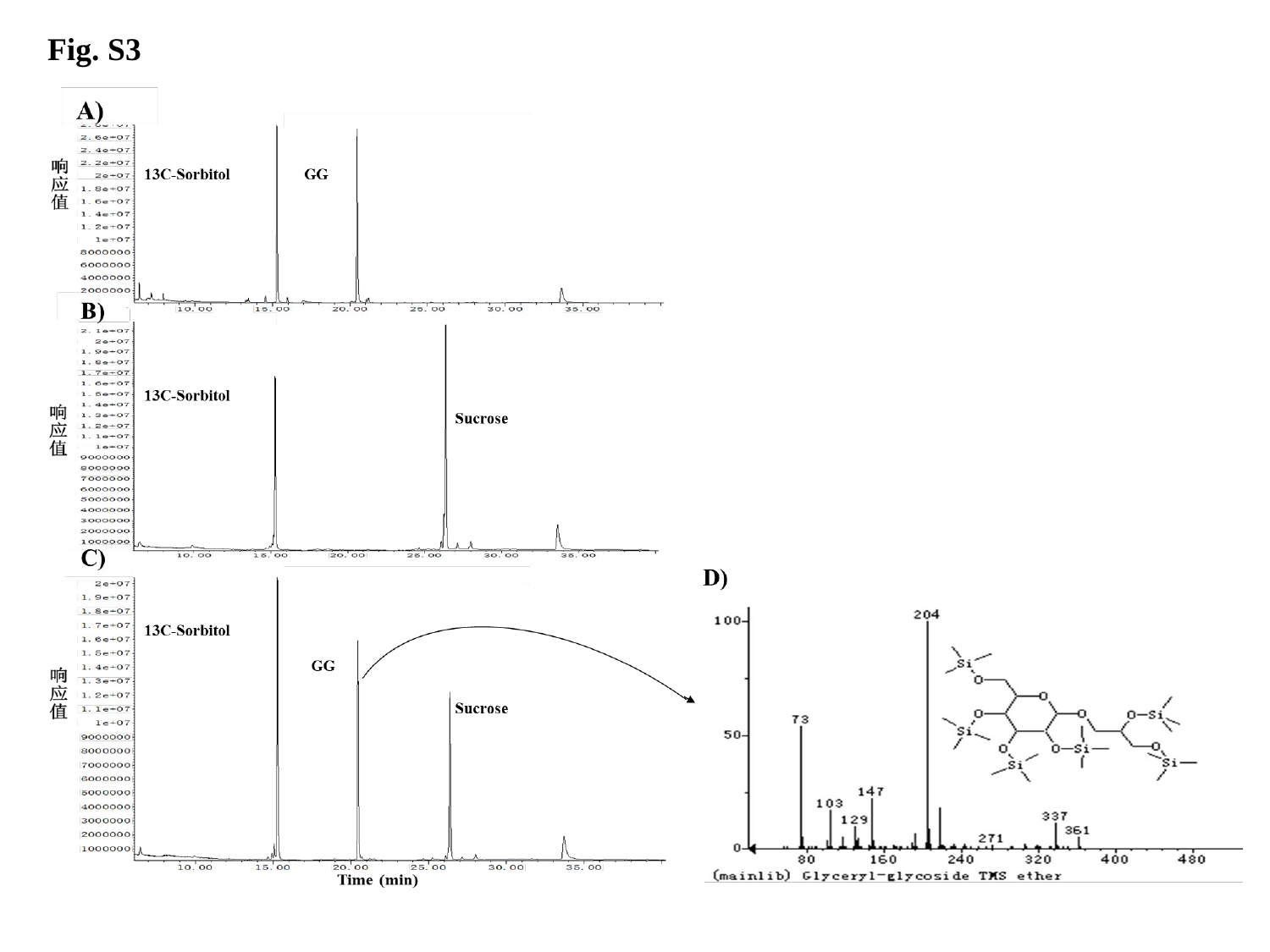

Fig. S3

## Slide 4
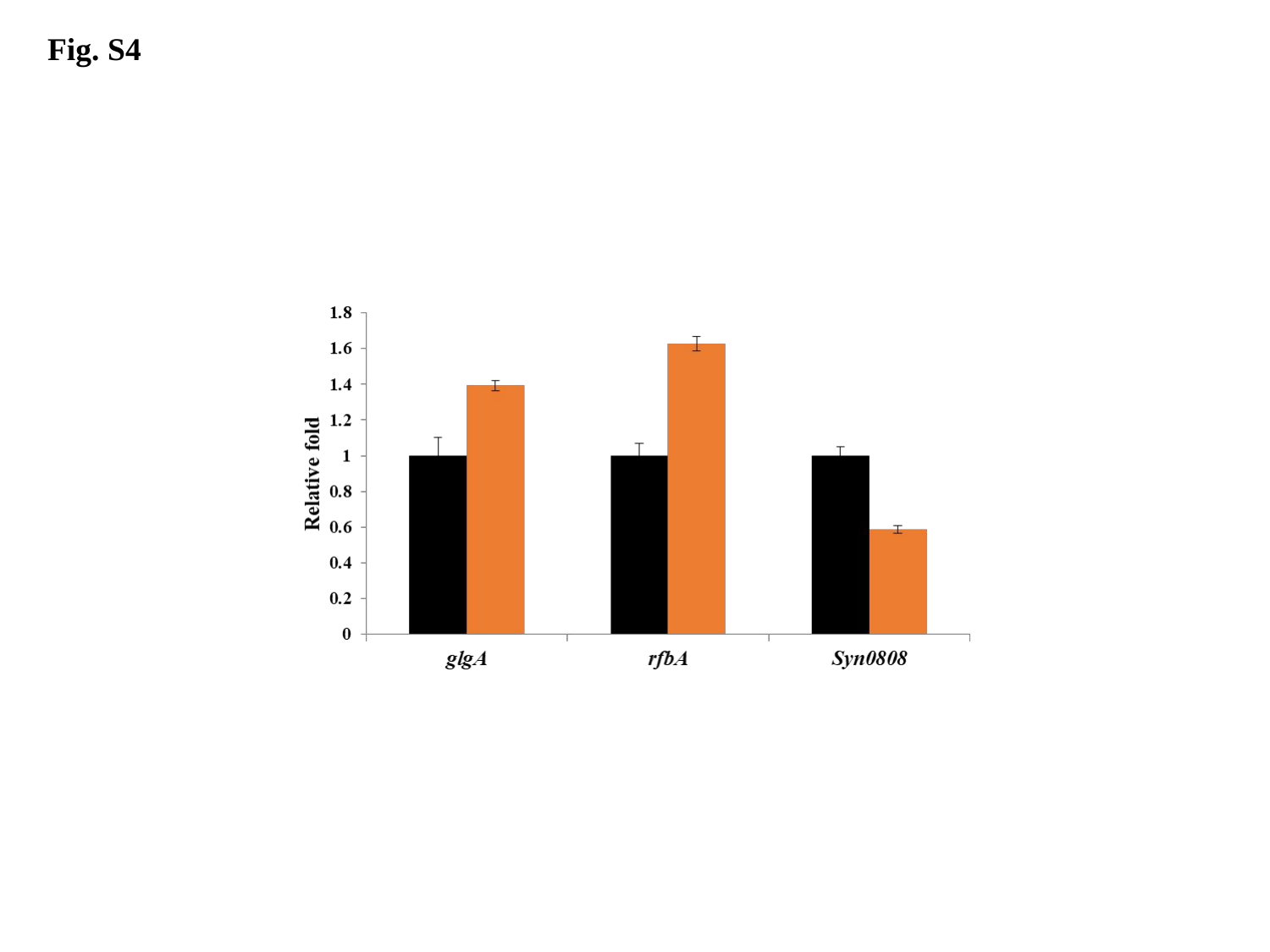

Fig. S4

## Slide 5
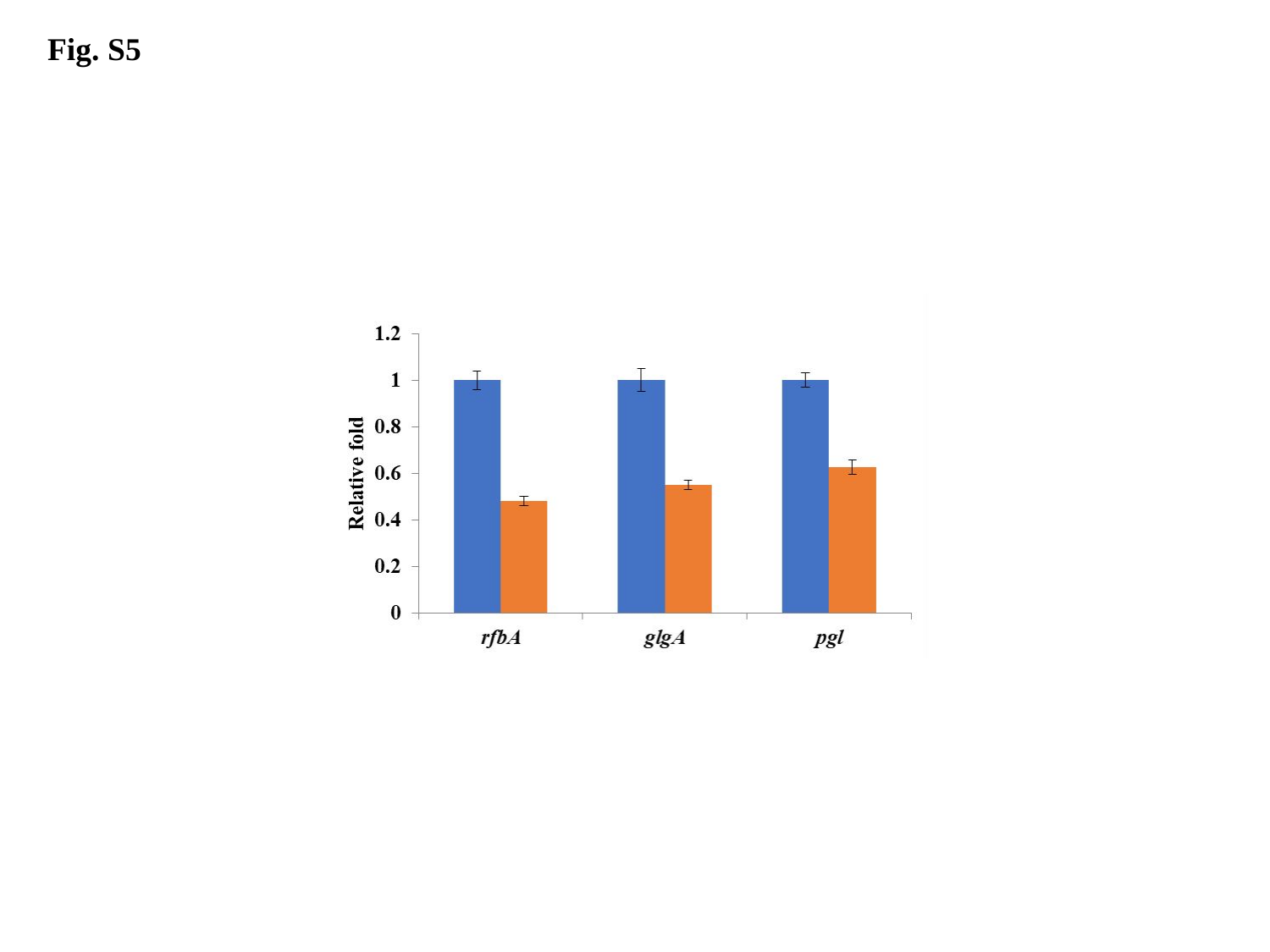

Fig. S5

## Slide 6
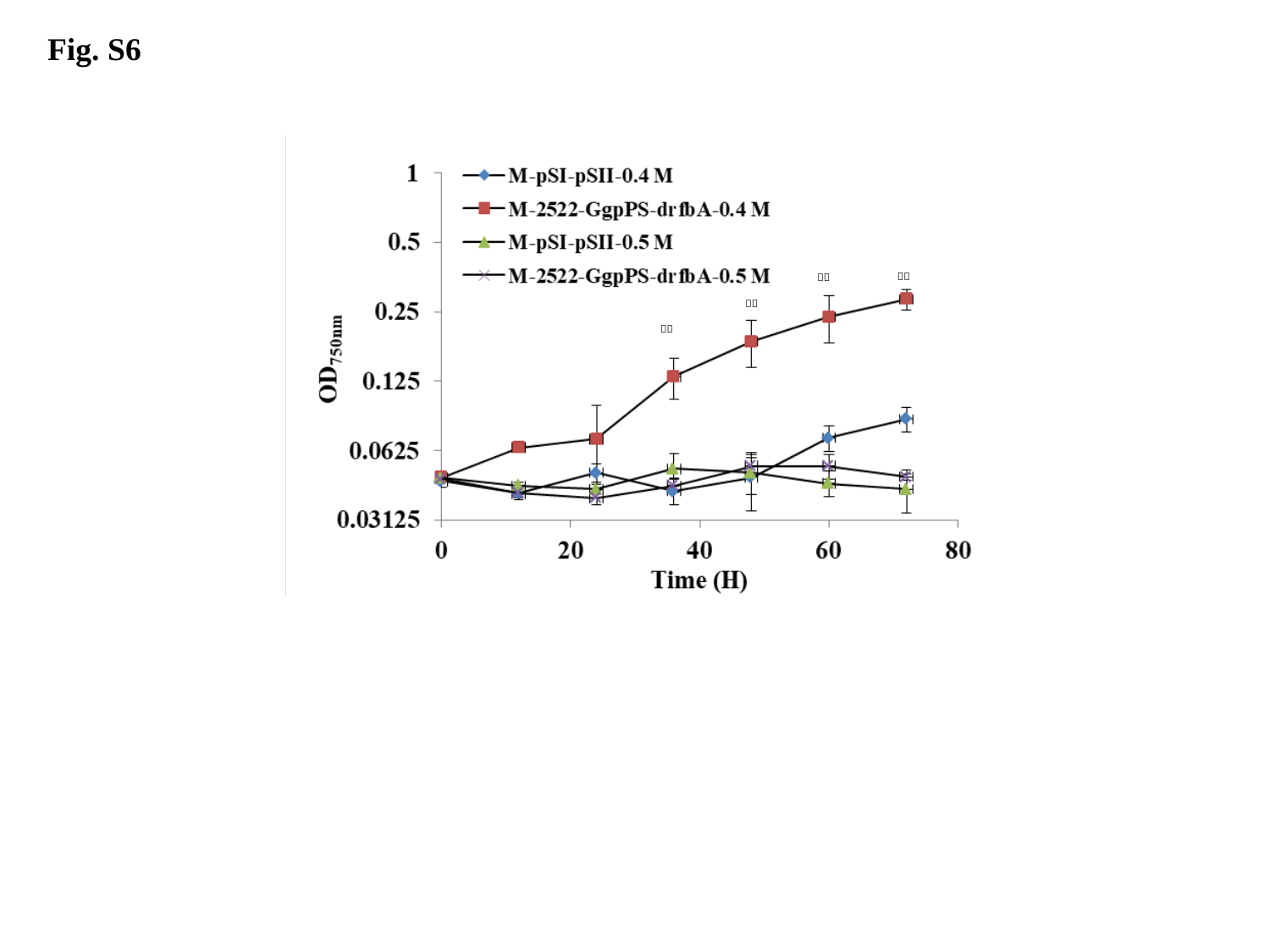

Fig. S6





Supplement: Supplementary Figure 1 — Identification of correct strains by colony PCR. (A) Amplification of the fragment Ptrc-sll0746-Ptrc-sll1566-TrbcL from strains M-pSI (control strain) and M-GgpPS; (B) amplification of the fragment Pcpc560-Syn2522-Ptrc-sll0746-Ptrc-sll1566-TrbcL from strains M-pSI and M-2522-GgpPS; (C) amplification of the fragment asrfbA-micC-TrbcL-Pcpc560-hfq-TrbcL from strains M-pSI-pSII (control strain) and M-2522-GgpPS-drfbA. M, marker. [file Presentation_1.PPTX]
