# Supplementary material for: Salt-Tolerant Synechococcus elongatus UTEX 2973 Obtained via Engineering of Heterologous Synthesis of Compatible Solute Glucosylglycerol
Source: Front Microbiol. 2021 May 18;12:650217. doi: 10.3389/fmicb.2021.650217 (PMC8168540; doi:10.3389/fmicb.2021.650217)
Supplement: Supplementary Table 1 — Primers used in this study. [file Table_1.DOCX]

**Table S1 Primers used in this study.**

| Primers | Sequences（5’–3’） |
| --- | --- |
| *sll1566*-F | GAGGTCGACATGAATTCATCCCTTGTGATC |
| *sll1566*-R | GAGGCGGCCGCCTACATTTGGGGGGGCTCTC |
| *sll0746*-F | GAGGTCGACATGGTATTACACCAACAACG |
| *sll0746*-R | GAGGAGCTCCTACTGGGAAAAATGGACTC |
| T*rbcl-*F | ACCGGTGTTTGGATTGTCGG |
| T*rc-*R | TGTGTGAAATTGTTATCCGCTCAC |
| RT*-glgA*-F | CGACACTCTTGATAAACGACTGAAC |
| RT*-glgA*-R | ACCTCAAGCCCCATCTCCTT |
| RT*-rfbA*-F | ATCTGGCACTCGGCTCTATCC |
| RT*-rfbA*-R | AGACCGGCAGCAGCTGTT |
| RT*-Syn7942-0808*-F | GCCATACGGCATCCCTGTT |
| RT*-Syn7942-0808*-R | AGACGGCTTTGTTCCTCCAA |
| T*rc*-F | ATGAGCTGTTGACAATTAATC |
| *7942-2522-*F | ATGCAAGAGGCAAAAGTTGCG |
| *7942-2522-*R | TCAGACCAATTCCGCTTTCAG |
| *sll1085-*F | ATGCGTAATTTCCCAGAAATCC |
| *sll1085-*R | TACGTAATCGATGCCTGATAGA |
| *slr1755-*F | ATGTCCTTTGCTAATGCCGAT |
| *slr1755-*R | TTAAAGATCAAAATCTTCAAATTC |
| *glpd1*-F | ATGTCTGCTGCTGCTGATAG |
| *glpd1*-R | CTAATCTTCATGTAGATCTA |
| Cpc560*-*R | TGAATTAATCTCCTACTTGAC |
| T*rbcl-*R | GCTGTCGAAGTTGAACATCAG |
| *sll1566*-R | CTACATTTGGGGGGGCTCTC |
| Cpc560*-*F | ACCTGTAGAGAAGAGTCCCTG |
| P*sbA2M-*F | TATCAATAAGTATTAGGTATA |
| Hfq*-*R | TTATTCGGTTTCTTCGCTGTC |
| Cat-R | TGATCGGCACGTAAGAGGTTC |
| As*glgA*-micC-F | ggcagccacgaacagaatccgcatTTTCTGTTGGGCCATTGCAT |
| As*rfbA*-micC-F | gatgccgcgccgtgcctcggtcatTTTCTGTTGGGCCATTGCAT |
| As*pgl*-micC-F | caagacagattgatgaacagacatTTTCTGTTGGGCCATTGCAT |
| PsbA2M-R | ATGTATTTGTCGATGTTCAG |
| RT*-glgA*-F | CGACACTCTTGATAAACGACTGAAC |
| RT*-glgA*-R | ACCTCAAGCCCCATCTCCTT |
| RT*-rfbA*-F | ATCTGGCACTCGGCTCTATCC |
| RT*-rfbA*-R | AGACCGGCAGCAGCTGTT |
| RT*-pgl*-F | GCCATACGGCATCCCTGTT |
| RT*- pgl*-R | AGACGGCTTTGTTCCTCCAA |
