# Supplementary material for: Salt-Tolerant Synechococcus elongatus UTEX 2973 Obtained via Engineering of Heterologous Synthesis of Compatible Solute Glucosylglycerol
Source: Front Microbiol. 2021 May 18;12:650217. doi: 10.3389/fmicb.2021.650217 (PMC8168540; doi:10.3389/fmicb.2021.650217)
Supplement: Supplementary Table 2 — The raw metabolomics data of the heatmap. [file Table_2.DOCX]

Table. S2: The raw metabolomics data of the heatmap.

| **Metabolites** | **M-pSI-pSII-1** | **M-pSI-pSII-2** | **M-pSI-pSII-3** | **M-2522-GgpPS-drfbA-1** | **M-2522-GgpPS-drfbA-2** | **M-2522-GgpPS-drfbA-3** |
| --- | --- | --- | --- | --- | --- | --- |
| NADPH | 29 | 24 | 20 | 50 | 50 | 45 |
| NADP | 3318 | 3615 | 2270 | 5725 | 5442 | 6822 |
| NAD | 726 | 996 | 736 | 582 | 661 | 699 |
| ADP-GLC | 100 | 110 | 123 | 150 | 134 | 163 |
| UDP-GLC | 578 | 854 | 699 | 540 | 432 | 667 |
| ATP | 2162 | 2111 | 1946 | 2318 | 2192 | 2378 |
| ADP | 1943 | 1817 | 1518 | 2367 | 2573 | 2829 |
| AMP | 5764 | 5814 | 4828 | 6351 | 6715 | 8226 |
| FBP | 323 | 294 | 336 | 264 | 306 | 305 |
| RiBP | 878 | 1000 | 723 | 1250 | 1511 | 1468 |
| F6P | 5687 | 5744 | 5137 | 4584 | 4819 | 5473 |
| R5P | 636 | 424 | 784 | 735 | 761 | 640 |
| E4P | 1133 | 921 | 910 | 1330 | 1374 | 1720 |
| CIT | 1788 | 1352 | 1481 | 2281 | 2442 | 2370 |
| 3PG | 560 | 626 | 560 | 3709 | 3704 | 4177 |
| 2PG | 369 | 318 | 244 | 1561 | 1853 | 1670 |
| G3P | 508 | 436 | 389 | 1332 | 1352 | 1625 |
| GAP | 518 | 434 | 341 | 497 | 518 | 611 |
| PEP | 2824 | 2102 | 2772 | 4229 | 4667 | 5912 |
| GLU | 1519 | 1499 | 1437 | 1618 | 1837 | 2071 |
| AKG | 274 | 225 | 199 | 283 | 276 | 316 |
| MAL | 1724 | 1504 | 1416 | 1699 | 1877 | 1817 |
| FUM | 2223 | 1599 | 2211 | 2098 | 1986 | 1903 |
| PYR | 1234 | 1097 | 1037 | 2397 | 2686 | 3072 |
| Sucrose | 122321 | 102321 | 132321 | 53442 | 43554 | 55432 |
